# Comparison of systolic and diastolic 3D SSFP for arterial dimensions and coronary artery origins in patients with congenital heart disease

**DOI:** 10.1186/1532-429X-14-S1-P110

**Published:** 2012-02-01

**Authors:** Ruchira Garg, Carlos F Rosental, Juan C Muniz

**Affiliations:** 1 Pediatrics, Division of Cardiology, Miami Children's Hospital, Miami, FL, USA; 2 Pediatric Cardiology, Juan P Garrahan Children's Hospital, Buenos Aires, Argentina

## Background

3D steady-state free precession (SSFP) sequences allow for acquisition of isotropic datasets without the need for IV placement and contrast administration. Similar to MR angiography, they can be post-processed using multiplanar reconstruction, allowing for cross-sectional vessel measurements. They are also ECG-gated, allowing for visualization of coronary artery origins, which cannot be reliably evaluated by MRA. Arteries are preferentially measured in systole to obtain maximal diameter. However, because of motion blurring, this may be a suboptimal phase for diagnosis of coronary origins.

We hypothesized that systolic SSFP (sSSFP) would provide larger measurements of arterial vessel diameter than either diastolic SSFP (dSSFP) or MRA sequences, yet provide adequate image quality for diagnosis of coronary artery origins.

## Methods

A retrospective review of 24 congenital heart disease patients with MRA, sSSFP and dSSFP datasets was performed. Cross-sectional measurements were performed in the mid-ascending aorta (AAo), right pulmonary artery (RPA) and left pulmonary artery (LPA). Diagnostic quality of SSFP datasets was determined for the LCA and RCA on a scale of 1-5.

## Results

**Figure 1:**
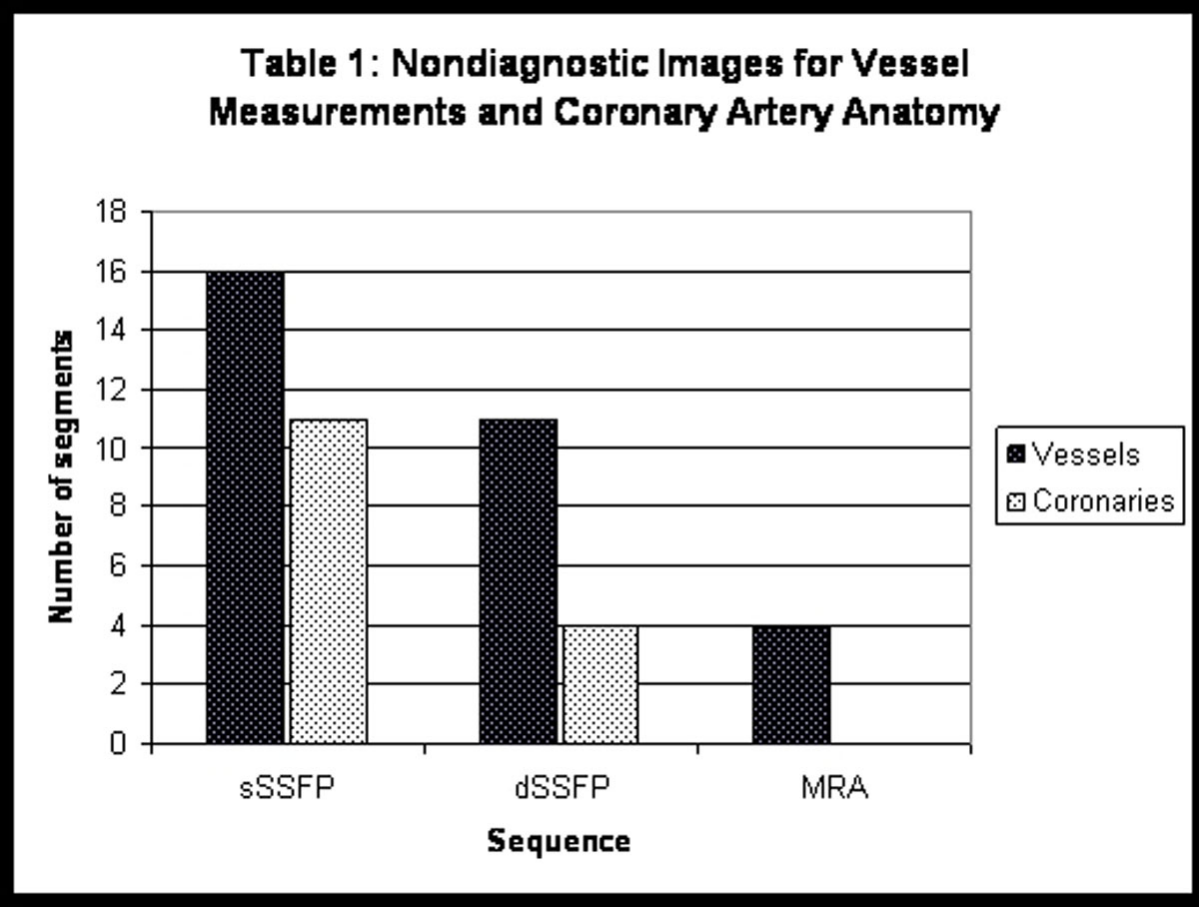


**Figure 2:**
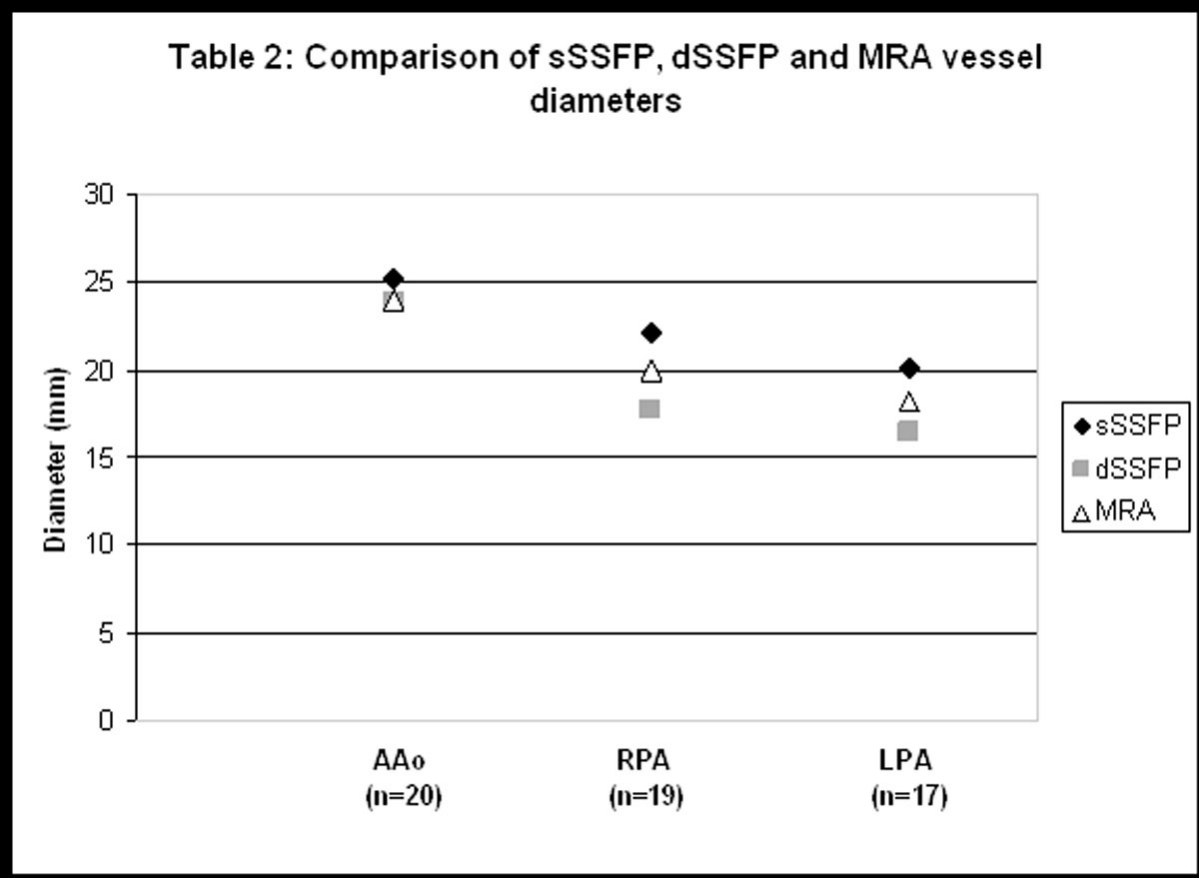


The mean age was 16.2 years (range 3.8-37.7) and mean weight was 46.9 kg (range 12.5-81). sSSFP sequences were nondiagnostic in 16 of 72 vessel segments, compared to 11/72 for dSSFP and 4/72 for MRA (Figure 1). Vessel comparisons were only performed on segments with adequate measurements on all 3 sequences (Figure 2). The mean AAo dimension was significantly larger for sSSFP (25.1mm) than either dSSFP (23.9mm,p=0.005) or MRA (24.0mm,p=0.01). The RPA was largest on sSSFP (22.1mm), followed by MRA (20mm,p<0.01) and dSSFP (17.7mm,p<0.01). Similarly, the LPA was largest on sSSFP (20.1mm), followed by MRA (18.2mm,p=0.03) and dSSFP (16.4mm,p<0.01).

For coronary evaluation, image quality was nondiagnostic (score <3) in 11/48 origins by sSSFP compared to 4/48 origins by dSSFP (Table 2). The mean LCA quality score was higher for dSSFP (4.1 vs 3.6,p=0.05); RCA scores were similar (3.9 vs 3.5,p=0.9).

## Conclusions

Systolic 3D SSFP yields larger vessel diameters than dSSFP or MRA. These measurements may be more reflective of true maximal diameter. However, poorer image quality of sSSFP precluded vessel measurement and diagnosis of coronary artery origins in a larger number of subjects compared with dSSFP or MRA.

## Funding

None.

